# Supplementary material for: A Humanized Yeast Phenomic Model of Deoxycytidine Kinase to Predict Genetic Buffering of Nucleoside Analog Cytotoxicity
Source: Genes (Basel). 2019 Sep 30;10(10):770. doi: 10.3390/genes10100770 (PMC6826991; doi:10.3390/genes10100770)
Supplement: Supplementary file 1 [file genes-10-00770-s001.zip › Additional_Files/Additional_File7_GOTermHeatmaps.zip!/Additional_File7_GOTermHeatmaps/positive_regulation_of_phosphorylation_of_RNA_polymerase_II_C-terminal_domain.pdf]

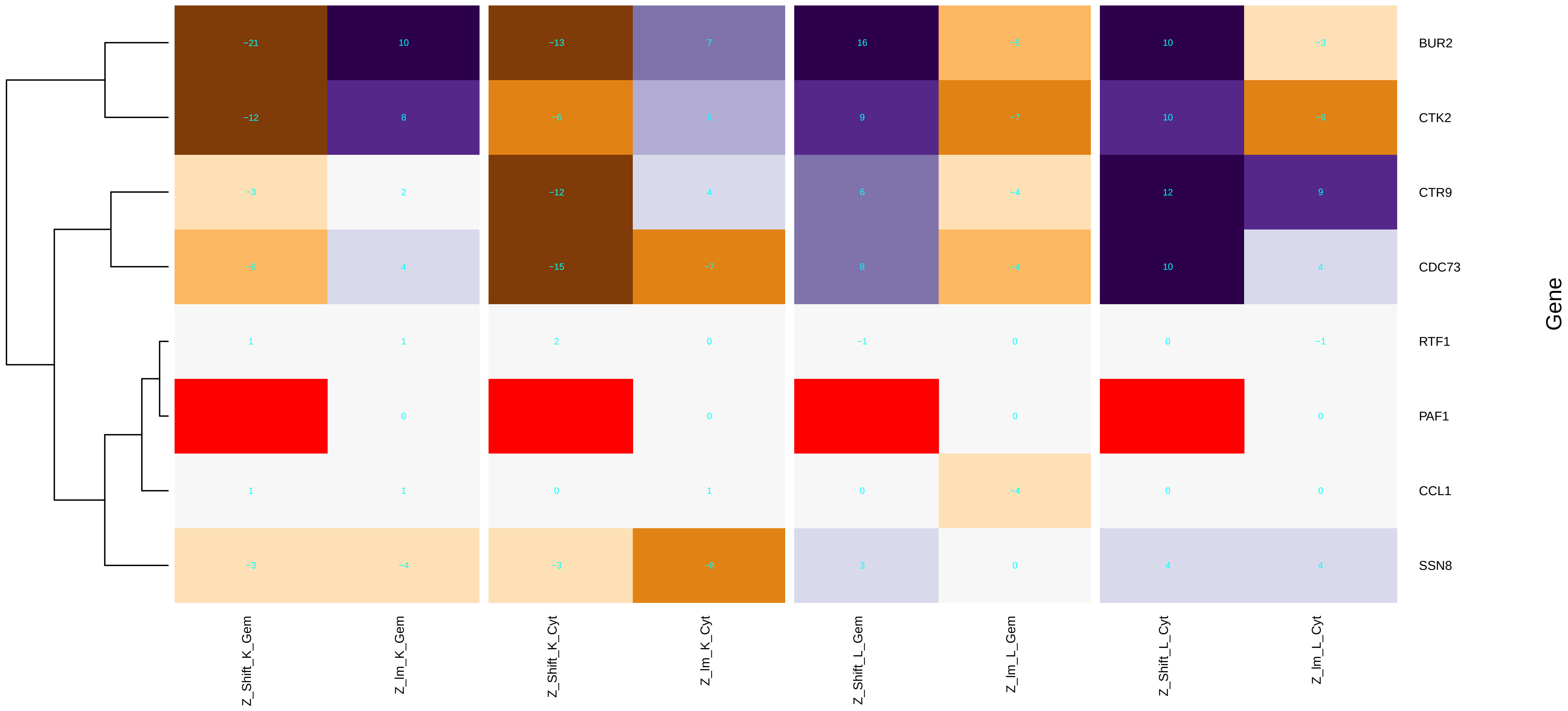
BUR2
CTK2
CTR9
CDC73
RTF1
PAF1
CCL1
SSN8
Gene
Z_Shift_K_Gem
Z_Im_K_Gem
Z_Shift_K_Cyt
Z_Im_K_Cyt
Z_Shift_L_Gem
Z_Im_L_Gem
Z_Shift_L_Cyt
Z_Im_L_Cyt
−21 10 −13 7 16 −5 10 −3
−12 8 −6 6 9 −7 10 −6
−3 2 −12 4 6 −4 12 9
−6 4 −15 −7 8 −4 10 4
1 1 2 0 −1 0 0 −1
0 0 0 0
1 1 0 1 0 −4 0 0
−3 −4 −3 −8 3 0 4 4
